# Genome-wide resequencing of KRICE_CORE reveals their potential for future breeding, as well as functional and evolutionary studies in the post-genomic era

**DOI:** 10.1186/s12864-016-2734-y

**Published:** 2016-05-26

**Authors:** Tae-Sung Kim, Qiang He, Kyu-Won Kim, Min-Young Yoon, Won-Hee Ra, Feng Peng Li, Wei Tong, Jie Yu, Win Htet Oo, Buung Choi, Eun-Beom Heo, Byoung-Kook Yun, Soon-Jae Kwon, Soon-Wook Kwon, Yoo-Hyun Cho, Chang-Yong Lee, Beom-Seok Park, Yong-Jin Park

**Affiliations:** Department of Plant Resources, College of Industrial Sciences, Kongju National University, Yesan, 340-702 Republic of Korea; Legume Bio-Resource Center of Green Manure (LBRCGM), Kongju National University, Yesan, 340-702 Republic of Korea; Department of Industrial & Systems Engineering, Kongju National University, Cheonan, 330-717 Republic of Korea; Korea Atomic Energy Research Institute, Advanced Radiation Technology Institute, 29 Geumgu-gil, Jeongeup-si, Jeollabuk-do 580-185 Korea; University of Science and Technology, Radiation Biotechnology and Applied Radioisotope Science, 217 Gajungro Yuseong-gu, Daejeon, 305-350 Korea; Department of Plant Bioscience, College of Natural Resources and Life Science, Pusan National University, Milyang, 627-706 Republic of Korea; Seedpia, 85, Maesil-ro, Kwonsun-ku, Suwon, 441-882 Republic of Korea; The Agricultural Genome Center, National Acedemy of Agricultural Science, Rural Development Adiministration, Wanju, 565-851 Republic of Korea

**Keywords:** Whole-genome resequencing, Rice, Core collection, Germplasm, SNP, INDEL, GWAS

## Abstract

**Background:**

Rice germplasm collections continue to grow in number and size around the world. Since maintaining and screening such massive resources remains challenging, it is important to establish practical methods to manage them. A core collection, by definition, refers to a subset of the entire population that preserves the majority of genetic diversity, enhancing the efficiency of germplasm utilization.

**Results:**

Here, we report whole-genome resequencing of the 137 rice mini core collection or Korean rice core set (KRICE_CORE) that represents 25,604 rice germplasms deposited in the Korean genebank of the Rural Development Administration (RDA). We implemented the Illumina HiSeq 2000 and 2500 platform to produce short reads and then assembled those with 9.8 depths using Nipponbare as a reference. Comparisons of the sequences with the reference genome yielded more than 15 million (M) single nucleotide polymorphisms (SNPs) and 1.3 M INDELs. Phylogenetic and population analyses using 2,046,529 high-quality SNPs successfully assigned rice accessions to the relevant rice subgroups, suggesting that these SNPs capture evolutionary signatures that have accumulated in rice subpopulations. Furthermore, genome-wide association studies (GWAS) for four exemplary agronomic traits in the KRIC_CORE manifest the utility of KRICE_CORE; that is, identifying previously defined genes or novel genetic factors that potentially regulate important phenotypes.

**Conclusion:**

This study provides strong evidence that the size of KRICE_CORE is small but contains high genetic and functional diversity across the genome. Thus, our resequencing results will be useful for future breeding, as well as functional and evolutionary studies, in the post-genomic era.

**Electronic supplementary material:**

The online version of this article (doi:10.1186/s12864-016-2734-y) contains supplementary material, which is available to authorized users.

## Background

Rice (*Oryza sativa* L.) is one of the most important staple crops in the world, providing a primary energy source for more than half of the world’s population [38]. It is closely associated with economic and political stability in many developing countries, such as Asia and Africa [38]. Moreover, the amount of land suitable for agriculture is decreasing due to a variety of factors such as rapid climate changes and industrialization, while rice-eating human populations continue to grow [38]. To meet the global nutritional and socio-economic demands, dedicated efforts towards developing superior rice varieties need to be reinforced, such as accumulating and combining beneficial alleles [14, 27, 28, 37].

Rice germplasm collections continue to grow in number and size around the world [22, 35]. The International Rice Research Institute (IRRI) holds more than 11,000 accessions in the collections [22]. Since maintaining and screening such massive resources remains challenging, a significant portion of beneficial alleles in wild relatives and landraces remain under-utilized [7, 12, 22, 28, 35]. Thus, it is important to establish efficient methods to discover and exploit these unused novel alleles to maximize rice-breeding efforts [27, 28, 35].

Developing core collections has been proposed to simplify germplasm conservation and promote their effective utilization [7, 28, 31, 44]. A core collection or set refers to a subset that represents the genetic diversity of an entire genetic resource of a species [7]. A good core set minimizes redundant entries while preserving the majority of available genetic diversity of the entire collection [12], in which 10 % of the entire collection generally constitutes the core collection [7, 12]. However, if the size of the whole collection is too large, a core collection still becomes unmanageable [7, 12]. The mini core collection (about 10 % of the core) can then be subsequently developed from the core using neutral molecular markers to achieve genetic comprehensiveness [7, 12, 39].

The advent of draft genome sequences of two rice subspecies, *O. sativa* ssp. *japonica* (Nipponbare) and ssp. *indica* (93–11), along with subsequent completion of high-quality *indica, japonica*, and *aus* reference genomes has accelerated rice functional genomics research [19, 28]. In addition, these reference sequences serve as frameworks for whole-genome resequencing, which is accomplished by alignments of short sequence reads produced by the next-generation sequencing (NGS) technology [18, 20, 28, 51]. Recently, applications of genome resequencing are rapidly expanding toward various rice natural resources, providing the crop research community with unprecedented genomic resolution and scale, as well as relevant functional diversity accumulated in the rice germplasm [18, 20, 28, 51]. Under these circumstances, resequencing the germplasm core collections would be beneficial to the related community.

Here, we report the whole-genome resequencing of the 137 rice mini core collection, potentially representing 25,604 rice germplasms in the Korean genebank of the Rural Development Administration (RDA). Based on the Nipponbare reference genome, our resequencing data yielded more than 15 million (M) SNPs and 1.3 M INDELs. Phylogenetic and population analysis using 2,046,529 high-quality SNPs successfully assigned rice accessions to the relevant rice subgroups, suggesting that the SNPs capture evolutionary signatures present in rice subpopulations. We conducted genome-wide association studies (GWAS) on four agriculturally important traits including ‘grain pericarp color’, ‘amylose content’, ‘protein content’, and ‘panicle number’. Among the detected association peaks, some identified previously discovered genes, indicating that KRICE_CORE can be implemented in GWAS to indentify novel alleles underlying agricultural traits. These results strongly suggest that resequencing results of KRICE_CORE are crucial for future rice breeding, as well as functional and evolutionary studies, in the post-genomic era.

## Results

### Sequencing of the Korean heuristic rice core set

Of the 166 rice core set selected through a heuristic approach, 137 accessions that can flower in Chungcheong province, South Korea, were selected (Additional file 1: Table S1). The Korean rice core set (KRICE_CORE) included domestically adapted weedy and landrace rice and bred lines, as well as introduced lines from Africa, Europe, and America (Additional file 2: Figure S1) [25, 52].

We sequenced KRICE_CORE using the Illumina Hiseq 2000 or 2500 platform (Table 1). The sequencing quality of the raw reads was generally high (91.6 %) (Table 1 and Additional file 3: Table S2). We next mapped short reads back to the IRGSP 1.0 rice genome (Methods). The mapping rate was generally high (95.48 % in average), varying from 89.5 to 98.86 % (Table 1 and Additional file 3: Table S2). We did not observe any significant correlations between mapping rate and sequencing depth (Additional file 2: Figure S2), indicating that the poor mapping rate was not just derived from the low sequencing depth or errors. Thus, the relative differences in mapping rates may be due to sequence divergence between some accessions of KRICE_CORE and the reference sequence. Overall, effective mapping depth was 9X on average (Table 1).

**Table 1:** Summary of sequencing statistics for KRICE_CORE

Variation range	Sequence read	Clean read rate	Deduplication read	Deduplication rate	Mapping rate	Average depth
Max	59,334,970	95.1	54,800,574	99.4	98.9	13.9
Min	29,090,954	86.4	25,021,515	95.9	89.5	6.3
Average	39,753,733.9	91.6	36,006,200	98.8	95.5	9.0

### Rice genome variation

**Fig. 1:**
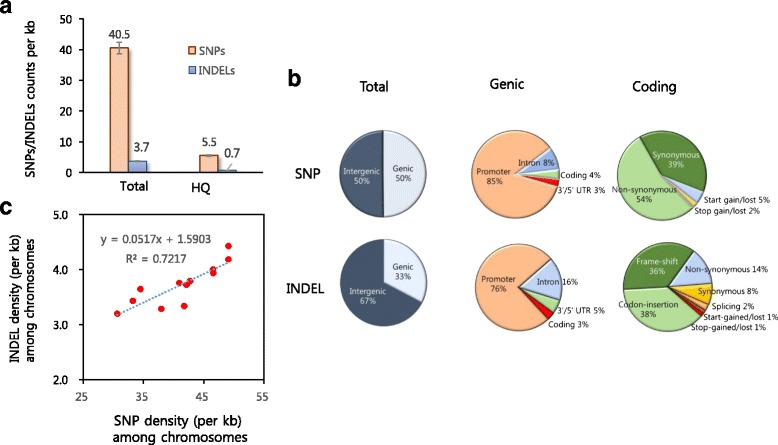
SNP and INDEL frequency in the KRICE_CORE population. **a** Comparison of the mean density of SNPs and Indels in KRICE_CORE. **b** SNP and INDEL distribution across various genome regions. The promoter of the genic region refers to the region 2 kb upstream of the transcription start site. **c** Correlation between SNPs and INDEL occurrences across KRICE chromosomes

We identified ~15 million (M) candidate SNPs from the 137 accessions (Table 2). About 13.5 % (2,046,529) of the SNPs did not contain missing data (hereafter referred to as high-quality SNPs [HQSNPs]; Additional file 4). We found that approximately 40.5 SNPs (5.5 by HQSNPs) were arrayed per kb across the KRICE_CORE genomes (Table 2 and Fig. 1a). We also found that 1.3 M INDELs existed in KRICE_CORE. Among those, 19.3 % were HQINDELs, showing an average density of INDEL per kb of 5.5 (0.7 for HQINDEL, respectively) (Table 2 and Fig. 1a).

**Table 2:** Summary of chromosomal SNP and INDEL distribution for KRICE_CORE

Chromosome	SNP				INDEL			
	Total		High quality		Total		High quality	
	Count	Density^a^	Count	Density	Count	Density	Count	Density
1	1,492,529	34.5	245,749	5.7	157,701	3.6	34,662	0.8
2	1,194,215	33.2	219,868	6.1	123,316	3.4	28,870	0.8
3	1,117,000	30.7	227,031	6.2	116,347	3.2	30,925	0.8
4	1,482,884	41.8	151,464	4.3	118,490	3.3	19,101	0.5
5	1,136,915	37.9	190,680	6.4	98,439	3.3	23,019	0.8
6	1,279,651	41.0	175,920	5.6	117,347	3.8	22,733	0.7
7	1,268,980	42.7	152,153	5.1	112,520	3.8	20,766	0.7
8	1,323,731	46.5	148,873	5.2	113,720	4.0	18,831	0.7
9	969,987	42.2	128,492	5.6	85,643	3.7	16,566	0.7
10	1,081,145	46.6	141,153	6.1	91,306	3.9	17,727	0.8
11	1,425,491	49.1	148,897	5.1	128,449	4.4	17,902	0.6
12	1,352,277	49.1	116,249	4.2	115,237	4.2	15,040	0.5
Total	15,124,805	40.5	2,046,529	5.5	1,378,515	3.7	266,142	0.7

^a^Density = SNP/INDEL count/length of the chromosome

About 50 % of SNPs were located in genic and intergenic regions, respectively (Fig. 1b and Additional file 5: Table S3). The majority of genic SNPs were distributed in the promoter region (85 %), followed by intronic (8 %), coding (4 %), 5′/3′UTR (3 %), and splicing (0.02 %) regions (Fig. 1b and Additional file 5: Table S3). In the coding region, we found that SNPs causing non-synonymous amino acid changes (non-synonymous SNPs) were enriched (54 %) (Fig. 1b and Additional file 5: Table S3). The ratio of nonsynonymous to synonymous SNPs was 1.38 (54 % vs. 39 % of coding SNPs), which was similar to previous reports [1, 2].

A lower abundance of INDELs is located in genic regions (33 %) (Fig. 1b and Additional file 5: Table S4). In addition, codon-insertion and frame-shifts are the most abundant classes of INDELs (38 and 36 %, respectively) in the coding region (Fig. 1b and Additional file 5: Table S4).

**Fig. 2:**
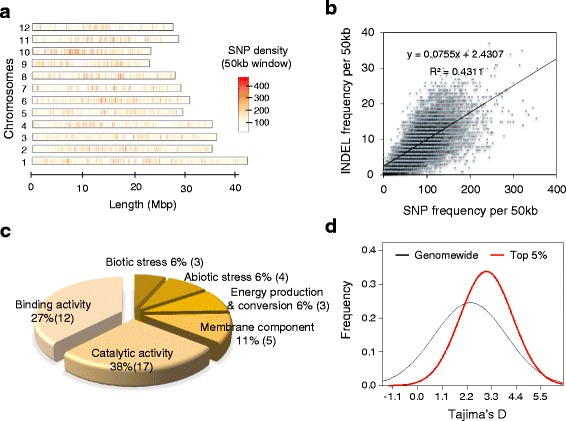
Genome-wide distribution of SNPs and INDELs of KRICE_CORE. **a** SNP density of the 50-kb window across the KRICE_CORE genome. **b** Correlation between SNP and INDEL occurrence across KRICE_CORE genome. **c** Functional category of genes in SNP/INDEL-enriched regions. **d** Distribution of Tajima’s D values of the genome wide and top 5 % SNP enriched regions

The density of SNPs and INDELs varied among rice chromosomes, ranging from 30.7 to 49.1 (4.2–6.4 with HQSNPs) and 3.2 to 4.2 (0.5–0.8 with HQINDEL), respectively (Table 2 and Additional file 5: Tables S3 and S4). We also found that the densities of SNPs and INDELs were positively correlated in chromosomes (Fig. 1c). To determine whether this correlation also occurred across local genomic regions, we investigated the correlation between SNP and INDEL densities within 50-kb windows across rice genomes (Fig. 2a). We found that SNPs are clustered in the rice genome (Fig. 2a), suggesting that SNPs were generated non-randomly across the rice genome [2, 15, 33, 47]. Furthermore, we observed a significant positive correlation between SNPs and INDELs across the rice genome, indicaing that INDELs are also clustered (Fig. 2b). Thus, the shared SNP and INDEL clustering patterns may be affected by a genome-wide set of regions that either commonly promote or restrict the genesis of SNPs and INDELs [2, 6].

We next investigated functional categories in the regions where SNP and INDEL frequencies were commonly high. Therefore, we performed gene enrichment tests for the top 5 % of regions. Among the genes functionally annotated, catalytic and binding activity were mostly enriched, followed by abiotic/biotic stress responses, energy production & conversion, and protein transport (Fig. 2c and Additional file 5: Table S5), which are important to maintain rice physiology. Considering that the mean Tajima’s D value for the windows of the top 5 % region was significantly higher than that at the genome-wide level, the accumulated SNPs and INDELs in these genes may facilitate creation of functional diversity (Fig. 2d).

### Population structure and genetic diversity of KRICE_CORE

**Fig. 3:**
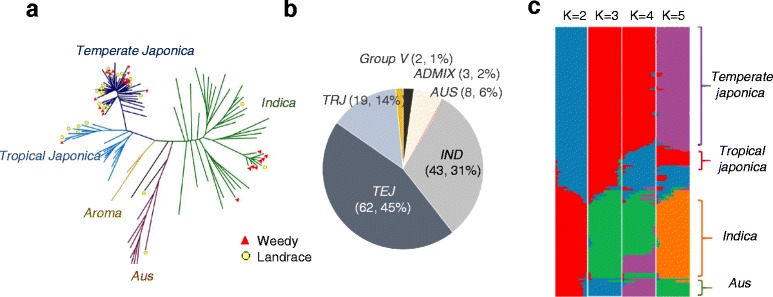
Population structure of KRICE_CORE. **a** Neighbor-joining analysis among KRICE_CORE using 2,046,529 HQSNPs. Landraces and weedy accessions are denoted as red triangles and yellow circles in the tree, respectively. **b** Pie graph designating the proportion of each subgroup. **c** Population structure analysis using FRAFFE. Each color represents one population. Each accession is represented by a horizontal bar, and the size of each colored segment in each horizontal bar represents the proportion contributed by ancestral population. K value represents the number of assumed clusters or populations

Using 2,046,529 HQSNPs, we performed population genetic studies to explore the genetic structures of KRICE_CORE. First, we constructed a neighbor-joining tree (Fig. 3a). To gain legitimate evolutionary insights from the resultant phylogenetic subdivisions, we incorporated the previously defined rice accessions into the tree analysis (Additional file 2: Figure S3) (Methods) [51]. We found that our tree analysis separated into five major subgroups, which partitioned into three major groups corresponding to japonica (81 accessions, 59 %), *indica* (hereafter *IND*, 43, 31 %) and *aus* (*AUS, 6 %*), with a further subdivision of *japonica* into *temperate* (*TEJ*, 62, 45 %) and *tropical* (*TRJ*, 19, 14 %) rice groups (Fig. 3a, b and Additional file 2: Figure S3). However, KRICE_CORE does not reflect *Group V* since only two accessions belong to the sub group (Fig. 3a, b and Additional file 2: Figure S5).

We found that Korean landrace and weedy accessions are mostly present in *TEJ* groups (Fig. 3a). Note that some are assigned to different subgroups, such as *IND*, *AUS* and *TRJ* (Fig. 3a). This led to the hypothesis that independent domestications of *TRJ* type together with *IND* (and others) have occurred in the Korean peninsula. However, these results may be derived from the outcrossing between weedy rice and *IND and TEJ* type rice varieties (Tongil type), which are widely cultivated in Korea. Since we have limited evidence for indica landrace rice, further studies are required to support this hypothesis.

We further investigated plausible population structures using FRAPPE, which estimates individual ancestry and admixture proportions assuming that K populations exist based on a maximum likelihood method (Fig. 3c). We analyzed the data by increasing K (the number of population) from 2 to 5 (Fig. 3c). For K = 2, we observed a division between *O. rufipogon*/japonica and *O. nibara*/others, including *IND* type. When K = 3, *AUS* subgroups were clearly separated from the *IND* group. When, K = 4, *TRJ* and *TEJ* were separated. When K = 5, a new subgroup including *group V* emerged from *TRJ* (Fig. 3c). These results supported the previous hypothesis that the two cultivated rice subspecies have been domesticated independently, forming two ancient populations; one population that gave rise to *indica* and the other to *japonica*, respectively. Since we independently confirmed that *O. rufipogon* and *O. nivara* were assigned to *japonica* and *indica* subgroups, we further supported the hypothesis that one population that gave rise to *indica* may have evolved from an ancestral population closely related to *O. nivara*, while the other that gave rise to *japonica* may have originated from *O. rufipogon* [51].

**Fig. 4:**
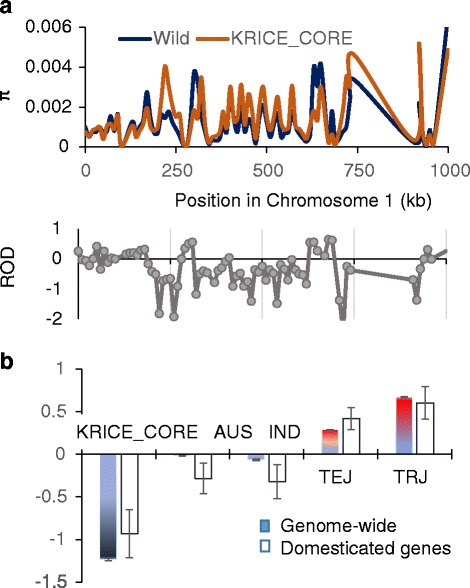
Genetic diversity of KRICE_CORE. **a** Nucleotide diversity (π) of wild rice (*O. rufipogan*) vs KRICE_CORE (*upper*) and the resulting ROD value (Methods). **b** Mean RODs among the subgroups in genome-wide or domesticated regions. Error bar indicates standard error of the mean (SEM). Sliding window analyses of π or ROD are shown for chromosome 1 with a 10-kb window

We estimated the sequence diversity of KRICE_CORE (Fig. 4 and Additional file 2: Figure S4). Reduction of diversity (ROD) was calculated from the sequence diversity (π) of KRICE_CORE and wild rice, *O. rufipogon,* across the entire rice genome (Fig. 4 and Additional file 2: Figure S4). We found that the ROD value of KRICE_CORE was highly dynamic across the rice genome, ranging from −30 to 1 (Fig. 4a and Additional file 2: Figure S4). Since HQSNPs were used in the analysis, it is possible that the majority of ROD values were correctly estimated. However, some RODs may be exaggerated, especially when π of wild rice was close to zero but those of others were higher.

We found that KRICE_CORE potentially retains a high diversity level (Fig. 4a, b, Additional file 2: Figure S4). Since *TRJ* is more closely related to *O. rufipogon* than *TEJ*, the average ROD value for *TRJ* of KRICE_CORE is much higher than that of *TEJ* (0.67 vs 0.29, *P* < 0.001 [one-way ANOVA]). However, incorporation of the divergent *AUS* and *IND* to KRICE_CORE, which decreased the ROD values greatly, significantly increased the overall diversity, as measured by ROD values (Fig. 4b). We also investigated ROD for regions that contain previously reported domesticated genes (Fig. 4b and Additional file 2: Figure S5). Considering that the amount of genetic diversity for the selected regions were comparable both at the genome-wide level and within each subpopulation (Fig. 4b), KRICE_CORE accommodates abundant functional diversities shaped from diverse external environments. These results will be useful for future rice breeding and fundamental studies in the post-genomic era to address current climate changes. Overall, these genetic and population structure results suggest that the size of KRIC_CORE is small but effectively retains genetic comprehensiveness.

### Genome-wide association studies (GWAS)

**Fig. 5:**
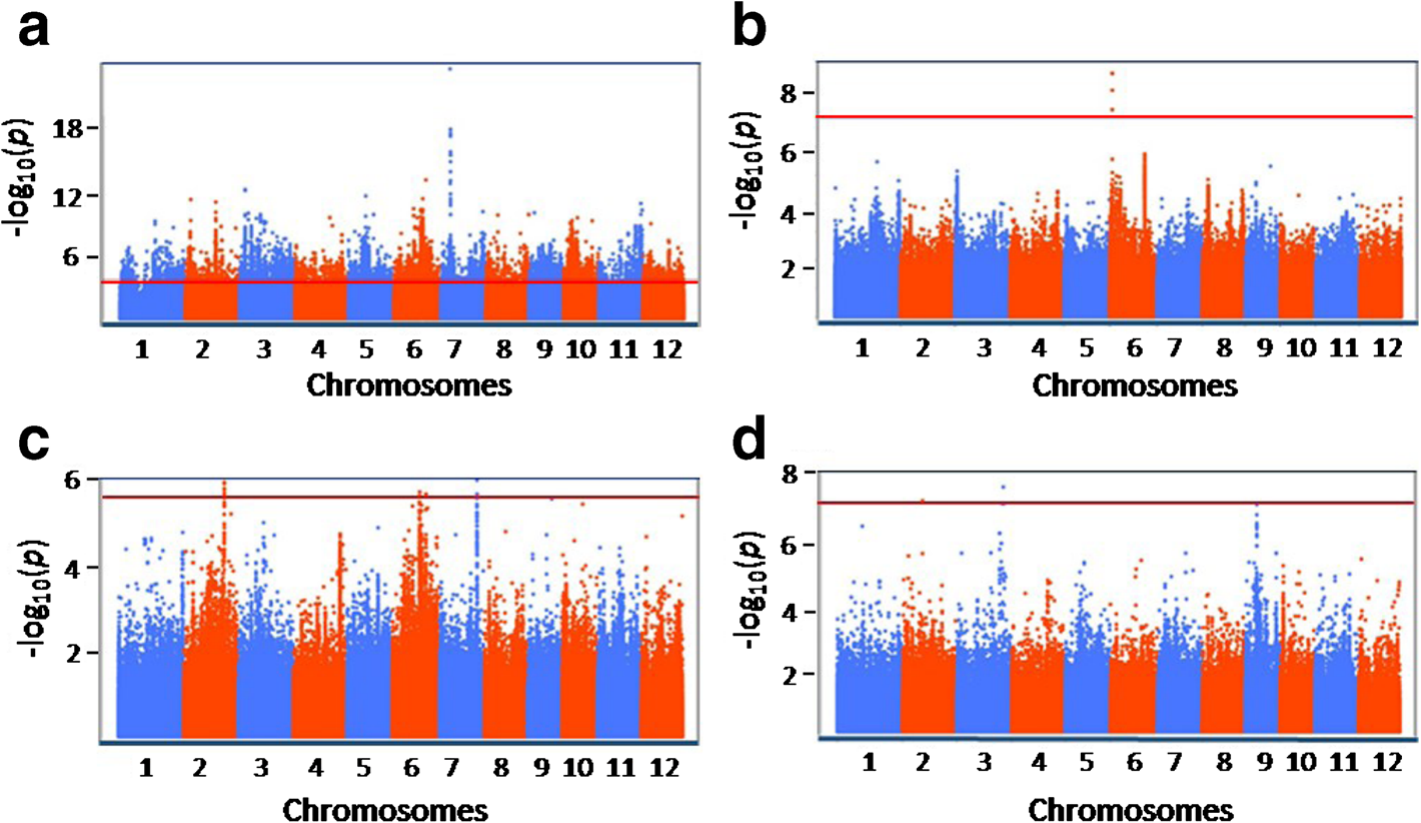
Genome-wide association studies of ‘pericarp color’ (**a**), ‘amylose content’ (**b**), ‘rice seed protein content’ (**c**), and ‘number of panicles per plant’ (**d**). Manhattan plots of the linear (for a) or mixed linear model (for **b**–**d**) are shown from negative log10-transformed *P-*values, which are plotted against the positions on each of the twelve chromosomes. A red horizontal line indicates the genome-wide significance threshold

We performed GWAS to determine whether we could exploit the natural diversity present in the KRICE_CORE to detect agriculturally useful alleles via association genetics (Fig. 5 and Additional file 2: Figure S6). We chose four agriculturally important phenotypes (‘pericarp color’, ‘amylose content’, ‘number of panicle per plant’, and ‘rice seed protein content’), which are representative of the qualitative or quantitative traits in KRICE_CORE (Fig. 5 and Additional file 2: Figure S6). We observed a wide range of phenotypic variation, spanning a 1.7- to 8.6-fold difference (Additional file 2: Figure S7). To determine whether the significant variabilities have a genetic basis, we employed a basic case/contro association test (for qualitative traits) using PLINK or a compressed mixed linear model (for quantitative traits) using GAPIT (Fig. 5 and Additional file 2: Figure S6) (Methods).

We found that GWAS signals associated with pericarp color and grain amylose percentage pinpointed the previously reported Rc locus at chromosome (ch) 7 and Waxy locus (at ch 6), respectively (Table 3, Fig. 5a, b, and Additional file 2: Figure S6). For the red pericarp color, the most significantly associated polymorphism (*P* = 3.05e-19) that explained ~46 % of the phenotypic variance was the 14 bp INDEL at 6,068,072 bp, which is located in the seventh exon of Rc (Table 3, Fig. 5a, and Additional file 2: Figure S6). For amylose content, one of the most significantly associated polymorphisms was located at the 5′ UTR region of the Wx locus (Os06g013300, at 1,765,761 bp of ch 6), explaining about 52 % of the phenotypic variation (Table 3, Fig. 5b, and Additional file 2: Figure S6).

**Table 3:** Genome-wide significant association signals of agronomic traits using the linear and compressed MLM

Traits	Ch	Position	Major allele (Count)	Minor allele (Count)	MAF^a^	R2	P value	Known Loci (Reference)
Pericarp color	7	6068072	A(95)	G(41)	0.30	0.465	3.05E-19	Rc(46)
	6	21304669	T(93)	C(43)	0.32	0.286	4.87E-11	
	3	3640060	G(116)	A(13)	0.12	0.268	2.48E-10	
	5	11479616	C(108)	T(26)	0.20	0.255	7.57E-10	
Amylose content	6	1765761	G(73)	T(61)	0.46	0.555	5.55E-09	Wx(21)
Protein content	7	24810573	A(77)	G(59)	0.43	0.348	1.14E-06	
	2	26791708	C(110)	T(25)	0.19	0.347	1.27E-06	
	6	18133739	G(93)	A(42)	0.31	0.339	2.15E-06	
Panicle number	3	30877559	C(128)	T(9)	0.07	0.404	3.13E-08	
	9	7760141	C(128)	T(()	0.07	0.386	1.09E-07	

^a^Minor allele frequency

**Fig. 6:**
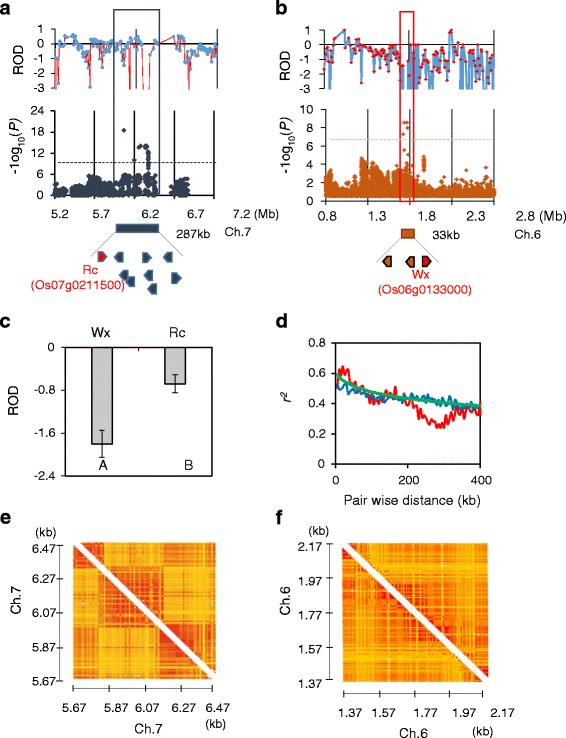
Regions of the associated signals near the Rc (**a**) and Wx (**b**) regions. The top of each panel shows the ROD for a 1-Mb window around the peak SNPs. Negative log_10_P-values for each SNP from the linear or compressed mixed linear model are plotted. Blue or dark orange dashed horizontal lines indicate the genome-wide significance cutoff for the Rc and Wx regions, respectively. The bottom of each panel within the blue or red box denotes the range of SNPs over the cutoff. **c** Mean ROD values for 400 kb around the Rc and Wx regions. **d** LD decay plots of the regions compared to genome-wide LD decay. **e** and **f** LD blocks of 400 kb on each side of the association peak for the Rc (**e**) and Wx (**f**) regions

We observed that the GWAS mapping precision varied between Rc and Wx regions. For pericarp color, the significantly associated SNPs were scattered around the 287-kb region of ch7 (Fig. 6a), but amylose content-related SNPs were dispersed within a 33-kb region (Fig. 6b). To determine whether the mapping accuracy was compounded by different evolutionary processes, such as specific domestication histories, we investigated the genetic diversity level and linkage disequilibrium (LD) structure across the 400-kb region around the association peaks (Fig. 6b–d; Methods). We found that the ROD score of the 400-kb region that included Rc (Rc region) was significantly higher than the Wx including the 400-kb region (Wx region) (mean ROD −0.68 (Rc region) vs-1.8 (Wx region), *P* < 0.001; one-way ANOVA), indicative of a significant reduction in the genetic diversity in the Rc region (Fig. 6a-c). This substantial genetic reduction of the Rc region in KRICE_CORE may be derived from the strong selection in favor of the 14-bp deletion since the domestication of the white grain rice. We also investigated LD decay across Rc and Wx regions (Fig. 6a–c). We found that the average LD decay of KRICE_CORE was about 85 kb. The LD decay of the Wx region was comparable, but the local LD pattern of the Rc region was atypical, indicating that the LD decay does not neutrally occur against the physical distance (Fig. 6d). To further examine the aberrant LD decay pattern over the Rc region, we constructed LD maps of Rc/Wx genomic regions (Fig. 6e–f). We found that obvious block-like LD structures exist in the Rc region, indicating that more powerful selective pressure might have acted on the Rc region (Fig. 6e, f). In addition, this extended LD structure may cause spurious associations in the Rc region, complicating prediction of the causative gene through GWAS mapping.

Several significant association signals were found for seed protein contents and panicle number (Table 3, Fig. 5, and Additional file 2: Figure S6). Although we could not identify any strong candidate genes for those traits, the novel SNPs identified here can be implemented in related breeding studies.

## Discussion

To address upcoming challenges for food production, the efficient use of rice germplasms is critical [13, 28]. These rice germplasms, adapted from diverse ecosystems, are valuable for rice breeders/researchers, potentially providing a broad array of useful alleles that enrich gene pools of current cultivated rice varieties [17, 19, 28]. Although *ex situ* conservation efforts are important for preserving diverse rice genetic resources, identifying novel and favorable genetic variants from the vast genebank collection remains challenging, requiring extensive screening processes [12, 14, 22, 35]. Therefore, the rice core collection is a powerful approach to accelerating utilization of exotic germplasms of the entire population [7, 31, 44]. In addition, the application of whole-genome resequencing technology would establish a potent platform for rapid forward genetic studies and genome-assisted breeding, which would facilitate discovery and exploitation of useful alleles [19, 28, 35].

### Genomic variations and population structure of KRICE_CORE

Here, we performed genome-wide resequencing of 137 KRICE_CORE populations originating from the 25,604 rice germplasms in the Korean genebank [52]. We identified ~15 million (M) candidate SNPs from the 137 KRICE_CORE (Table 2); 13.5 % (2,046,529) were HQSNPs at a 9x depth. Our neighbor-joining tree analysis using HQSNPs clearly showed that KRICE_CORE comprises the major rice subgroups, including *IND* together with *TEJ*, *TRJ,* and *Aus* (Fig. 3 and Additional file 2: Figure S3). Regarding the 3,000 rice genome analyses, approximately 75 % of the SNPs were covered by our study, although only 4 % of rice accessions were sequenced, suggesting that KRICE-CORE represents the diversity of the whole germplasm collection with relatively few accession numbers. These data indicate that the implementation of heuristic approaches using PowerCore software effectively reduced core entry but retained maximum diversity [25].

We found that many Korean landrace and weedy accessions were assigned to distinct ecotype groups (Fig. 3 and Additional file 2: Figure S3). Given that *TEJ* are dominant in the Korean peninsula, it is possible that *TRJ* and *IND* rice may have been introduced or domesticated historically. Recently, South Korea has faced gradual changes in many climate parameters, including annual temperature, rainfall amount, and precipitation [9]. The most significant climate change reported is a massive increase in the range of temperature fluctuations throughout the four seasons. This leads to heavier precipitation during the summer together with elevated temperature [24]. As a result, the Korean Peninsula is predicted to become a subtropical region [23]. To rapidly adapt to on-going climate change, these *IND* and *TRJ* weedy and landrace accessions acclimated in Korea would be valuable genetic resources. Still, many interesting evolutionary questions remain for these landrace and weedy lines, including their origins and transferring/fixation processes [42]. Addressing these questions could increase our understanding of the relevant domestication mechanisms, providing information for future improvements of Korean rice cultivars [42].

In KRICE_CORE, we observed a clear separation between *japonica* and *indica* type at *K = 2*. As the *K* value increased, *aus* diverged from the *indica* population. *Tropical* and *temperate* japonica types were separated from the *japonica* population (Fig. 3c). Since *O. rufipogon* and *O. nivara* are assigned to the *japonica* and *indica* at K = 2 (Fig. 3c and Additional file 2: Figure S3), respectively, we support the hypothesis that two cultivated rice subspecies, *indica* and *japonica*, may have originated from two ancient subpopulations [43, 51]; one population that gave rise to *indica* may have evolved from an ancestral population from *O. nivara* and the other for *japonica* may have originated from *O. rufipogon* [43, 51]. However, further studies are required to determine which model explains the actual domestication processes.

### Functional significance of SNPs and INDELs

It is expected that about 40 SNPs and 5.5 INDELs are arrayed per kb across the KRICE_CORE genome (Fig. 1a). However, we found that SNPs and INDELs are not evenly distributed. Rather, significant portions of SNPs and INDELs are clustered in the KRICE_CORE genome (Figs. 1c and 2a). Interestingly, the clustering patterns of those two genetic variants are strongly correlated (Fig. 2b), suggesting that SNPs and INDELs may arise non-randomly and such genesis mechanisms are highly connected [2, 6, 48]. These results are consistent with observations in mammalian and avian genomes [6, 48], indicative of the presence of mutation hot spots or cold spots across the genome [3]. The local recombination variability is reported as one of the key determinants for the mutation hot spot, driving sequence evolutions and genetic diversities [3]. Although high mutation rates may be an important driver underlying the co-clustering pattern, other evolutionary forces could create the non-randomness [2]; among these, natural selection can modulate local variability’s along a chromosome from responding to the ambient environments [11, 49]; balancing selection tends to create regions of increased variability [2, 8, 50], while purifying and directional selection tend to reduce variability [40]. Thus, the effect of mutagenic hot spots in population data may be obscured by a complex interplay of these factors [3]. Varela and Amos (2010) showed that INDEL locations are conserved in the same location between humans and chimpanzees, but SNP density appears highly variable; clusters found in chimpanzees are often not found at the same site in humans, indicating that a species dependent regional context or signature may regulate the surrounding SNP density following the genesis of INDEL [16, 30, 41, 48, 50]. Further studies should explore the major determinants causing SNP/INDEL clustering and its evolutionary consequences in plant/crop species.

We found that many genes in important biological processes, including, catalytic and binding activity, abiotic/biotic stress responses, and energy production are distributed in the high SNP regions (Fig. 2c and Additional file 5: Table S5). Given that the mean Tajima’s D value for the windows of the top 5 % genic SNP region is significantly higher than that at the genome-wide level (3.03 vs 2.36 in mean Tajima, *p* < 0.01 by bootstrapping method), it is believed that accumulated SNPs and INDELs in these genes are more or less likely to be beneficial in creating multiple alleles, potentially resulting in functional diversity. Thus, resequencing results of KRICE_CORE would increase our understanding of the molecular basis of the functional differences, especially related to important agricultural traits [1].

### Genome-wide association studies (GWAS)

GWAS has been implemented to efficiently identify candidate genes related to various useful agricultural traits in many crop species, including rice [19, 29]. Given that significant associations between genetic variations and phenotypic diversity does not require prior knowledge, GWAS with high genome coverage of SNP markers provides a genomics platform to dissect previously unknown adaptive genetic variation accumulated in plant germplasm resources over time [5]. After identifying candidate genes, GWAS allows for informed choice of parents for QTL analysis based on the haplotype information, along with suggesting targets for the following mutagenesis and transgenic studies [26]. Here, we conducted proof-of-concept GWAS analysis using four agriculturally important phenotypes (Fig. 5); the ‘pericarp color’ represents the qualitative trait of KRICE_CORE, while the others are for the quantitative traits.

We found that GWAS signals associated with pericarp color and grain amylose percentage pinpointed the previously reported Red pericarp (Rc) and Waxy (Wx) loci, respectively (Fig. 5). For the red pericarp color, the most significantly associated polymorphism (*P* = 3.05e-19) is the 14 bp INDEL, which is located in the seventh exon of Rc (Fig. 6a and Table 3). Sweeney et al. (2006) reported that the recessive rc allele by the 14-bp deletion nullifies the regulatory function in the proanthocyanidin synthesis pathway, preventing development of the pigmented pericarp layer [45, 46]. We found that the majority (>94 %, 80 out of 85) of white pericarp KRICE_CORE accessions contain a 14-bp deletion in exon 7 of the gene. The exceptional five white pericarp accessions that do not have the deletion may have been generated through rc-independent mechanisms. For amylose content, one of the most significantly associated polymorphisms is located in the 5′ UTR region of the Wx locus (Fig. 6b). Very close to the association peak, we identified a splice donor site in intron 2 of Wx. Previously, it was reported that Wx^b^, the GtoT point mutation at the site, is associated with the absence of amylose-characterizing glutinous rice varieties, suggesting that natural variations to the site create functional diversities for the amylose content [21]. Considering that the association peaks are found dominantly at Rc and Wx in KRICE_CORE and others [20], Rc and Wx largely underpin the red pericarp color and amylose content, respectively [20, 45, 46]. To improve the power to recover meaningful but minor associations (especially for amylose content), the sample size should be increased using diverse germplasms [4, 26, 34].

We observed that the GWAS mapping precision varies between Rc and Wx regions. For the pericarp color, the significantly associated SNPs are scattered around the 287-kb region of ch7, but amylose content-related SNPs are dispersed within the 33-kb region (Fig. 6a, b). We concluded that the much stronger LD pattern of Rc may complicate accurate prediction of the causative gene, as observed in other rice GWAS (Fig. 6e, f) [20, 45, 46]. Hence, it is believed that local LD estimations may be required when candidate genes are considered following GWAS. Besides, several significant novel associations were found for seed protein contents and panicle numbers. These results clearly demonstrate that GWAS implemented in KRICE_CORE offer a viable approach for dissecting the causal genetic variants of qualitative and quantitative phenotypes.

## Methods

### Genomic DNA library construction and sequencing

Quality control (QC) for prepared genomic DNA was performed; A_260/280_ > 1.6 and A_260/230_ > 1.6 for UV absorbance using Nanodrop (Tecan) and ≥5 ug fluorescence concentration using the Quant-iT BR assay kit (Invitrogen). DNA was run on 0.7 % agarose gels to assess degradation.

Genomic DNA was fragmented into approximately 100–300-bp insert sizes by sonication. Each DNA fragment was end-repaired using the Paired-End DNA Sample Prep Kit (Illumina; San Diego, CA, USA), followed by the addition of a 3′-A overhang and ligation of the adapters. Size-selection for DNA fragments of 200–300 bp was performed using a gel extraction kit (Qiagen). The constructed DNA samples were quantified using Quant-iT™ dsDNA High Sensitivity Assay Kit (Invitrogen; Carlsbad, CA, USA) on an Agilent 2100 Bioanalyzer (Agilent Technologies; Santa Clara, CA, USA). After qPCR validation, the resulting libraries were subjected to paired-end sequencing with a 100-bp read length using the Illumina HiSeq 2500 platform (Illumina). Raw image files were processed by Illumina Real-Time Analysis (RTA) for image analysis and base calling.

### Bioinformatics analysis

Raw sequences were first processed to obtain an average quality score (QS) per read ≤20 by trimming 3′-end of reads using SICKLE (https://github.com/najoshi/sickle). High-quality reads were aligned to the rice reference genome (IRGSP Build 5) using the Burrows-Wheeler Aligner (BWA) (version 0.7.5a) with default parameters [32]. Reads not meeting BWA quality criteria or not matching the reference genome were removed. PCR duplicate reads were removed using PICARD (version 1.88) (http://broadinstitute.github.io/picard/). Using the Genome Analysis Toolkit (GATK) (version 2.3.9 Lite) [36], regional realignment and quality score recalibration were performed, after which SNPs and InDels were identified with ≥3X read depth coverage. The resulting variants were annotated according to genomic position and overlap with current genome annotation using snpEff [10].

### Plant materials

Of the 166 accessions in the rice mini core set, selected from a composite population of 4,046 accessions using a heuristic approach (Additional file 2: Figure S1), 137 accessions that can flower in Chungcheong province, South Korea, were included in this study [25, 52].

### Population structure inference

To confirm the subgroups of our 137 accessions, we combined our 137 sequence and the published 50 sequence data, which have clear subgroup information, and then performed a phylogenetic study using the PHYLIP 3.68 (http://evolution.genetics.washington.edu/ phylip.html) with 100 replicates for a bootstrap confidence analysis. FigTree v1.4.0 (http://tree.bio.ed.ac.uk/software/figtree/) was used to plot the constructed tree. We used the same method to construct our 137 phylogenetic tree. MEGA6 [5] was used to present the phylogenetic tree.

We further used the maximum-likelihood-based software FRAPPE to investigate the population structure of the 137 accessions. We ran 10,000 iterations and the number of clusters (*K*) was considered from 2 to 5.

### Estimation of population parameters

Linkage disequilibrium parameters (*r*^*2*^) for estimating the degree of LD between pair-wise SNPs (58,008 high quality SNPs with physical distance >5 kb) was calculated using the software TASSEL 5.0 (http://www.maizegenetics.net/#!tassel/c17q9) with 1,000 permutations. The LD decay rate was measured as the chromosomal distance at which the average pairwise correlation coefficient (*r*^*2*^) decreased to half of its maximum value. Nucleotide diversity (π) and Tajima’s D were calculated by Vcftools [6] with a 50-kb slide window. The nucleotide diversity (*π*) is defined as the average number of nucleotide differences per site between any two DNA sequences chosen randomly from the sample population:

$$ \pi ={\displaystyle \sum_{ij}}{x}_i{x}_j{\pi}_{ij}={\displaystyle \sum_{i=1}^n}{\displaystyle \sum_{j=1}^i}{x}_i{x}_j{\pi}_{ij} $$

where x_*i*_ and x_*j*_ are the respective frequencies of the *I*th and jth sequences, *π*_*ij*_ is the number of nucleotide differences per nucleotide site between the *i*th and *j*th sequences, and *n* is the number of sequences in the sample. The summation is taken over all distinct pairs *i,j*, without repetition.

Tajima’s D test followed the method developed by Tajima (1989). At equilibrium between genetic drift and selectively neutral mutation, the expected value of *D* is close to zero.

To measure genetic diversity, we used the reduction of diversity (ROD) as:

$$ ROD=1-\frac{\pi_{cul}}{\pi_{wild}} $$

where *π*_*cul*_ and *π*_*wild*_ are the values of *π* for the cultivated and wild varieties, respectively.

### Genome-wide association analysis

For the qualitative trait (pericarp color), we performed GWAS using PLINK (http://pngu.mgh.harvard.edu/~purcell/plink/) software. For the other three quantitative traits (amylose content, protein content and panicle number), we performed GWAS using GAPIT [7] with a mixed linear model, where the PCA matrix and relative kinship matrix were included as fixed and random effects.

## Conclusion

This study provides strong evidence that the size of KRICE_CORE is small but contains high genetic and functional diversity across the genome. The genome information through the whole-genome resequencing technology would establish a potent platform for forward/reverse genetic studies as well as genomeassisted breeding, facilitating future discovery and exploitation of useful alleles from rice germplasms.

## Additional files

Additional file 1: Table S1. List of KRICE_CORE accessions used for whole-genome resequencing. (XLSX 15 kb) Additional file 2: Figure S1. Geographical (a,b) and methodological (c) origins of KRICE_CORE. **Figure S2.** Scatter plot between mapping rate and sequencing depth of KRICE_CORE. **Figure S3.** Neighbor-joining tree analysis of previously defined rice accessions. **Figure S4.** Sliding-window analysis of reduction of diversity (ROD) across KRICE_CORE genome. **Figure S5.** Genomic positions of previously identified domesticated genes. **Figure S6.** Quantile-quantile plot of the genome-wide association studies of ‘pericarp color’ (a), ‘amylose content’ (b), ‘rice seed protein content’ (c), and ‘number of panicles per plant’ (d). **Figure S7.** Phenotypic variation of the target traits for the GWAS study. (PPTX 1389 kb) Additional file 3: Table S2. Sequencing and mapping statistics for individual KRICE_CORE accessions. (XLSX 36 kb) Additional file 4: High-quality SNPs (HQSNPs) in KRICE_CORE from chromosomes 1–6. Individual HQSNPs (minor allele frequency >0.05) from chromosomes 1–6 of 137 KRICE_CORE accessions are included in the zip file chr1_6_KRICE_CORE_HQSNP.zip (TAB delimited txt file format). (ZIP 24253 kb) Additional file 5: Table S3. SNP distributions across various KRICE_CORE genomic regions. **Table S4.** INDEL distributions across various KRICE_CORE genomic regions. **Table S5.** Functional categories enriched in the high SNP/INDEL region across the KRICE_CORE genomes. (PPTX 54 kb) Additional file 6: HQSNPs of KRICE_CORE from chromosomes 7–12. Individual HQSNPs (minor allele frequency >0.05) from chromosomes 7–12 of 137 KRICE_CORE accessions are included in the zip file chr7_12_KRICE_CORE_HQSNP.zip (TAB delimited txt file format). (ZIP 17471 kb)

## Abbreviation

GWAS: Genome-wide association studies
HQSNPs: High-quality SNPs
IND: Indica
LD: Linkage disequilibrium
M: Million
NGS: Next-generation sequencing
non-synonymous SNPs: Non-synonymous amino acid changes
Rc: Red pericarp
RDA: Rural Development Administration
ROD: Reduction of diversity
SNPs: Single nucleotide polymorphisms
TEJ: Temperate japonica
TRJ: Tropical japonica
Wx: Waxy
