# Supplementary material for: Genome-wide resequencing of KRICE_CORE reveals their potential for future breeding, as well as functional and evolutionary studies in the post-genomic era
Source: BMC Genomics. 2016 May 26;17:408. doi: 10.1186/s12864-016-2734-y (PMC4882841; doi:10.1186/s12864-016-2734-y)
Supplement: Additional file 2: Figure S1. — Geographical (a,b) and methodological (c) origins of KRICE_CORE. Figure S2. Scatter plot between mapping rate and sequencing depth of KRICE_CORE. Figure S3. Neighbor-joining tree analysis of previously defined rice accessions. Figure S4. Sliding-window analysis of reduction of diversity (ROD) across KRICE_CORE genome. Figure S5. Genomic positions of previously identified domesticated genes. Figure S6. Quantile-quantile plot of the genome-wide association studies of ‘pericarp color’ (a), ‘amylose content’ (b), ‘rice seed protein content’ (c), and ‘number of panicles per plant’ (d). Figure S7. Phenotypic variation of the target traits for the GWAS study. (PPTX 1389 kb) [file 12864_2016_2734_MOESM2_ESM.pptx]

## Slide 1
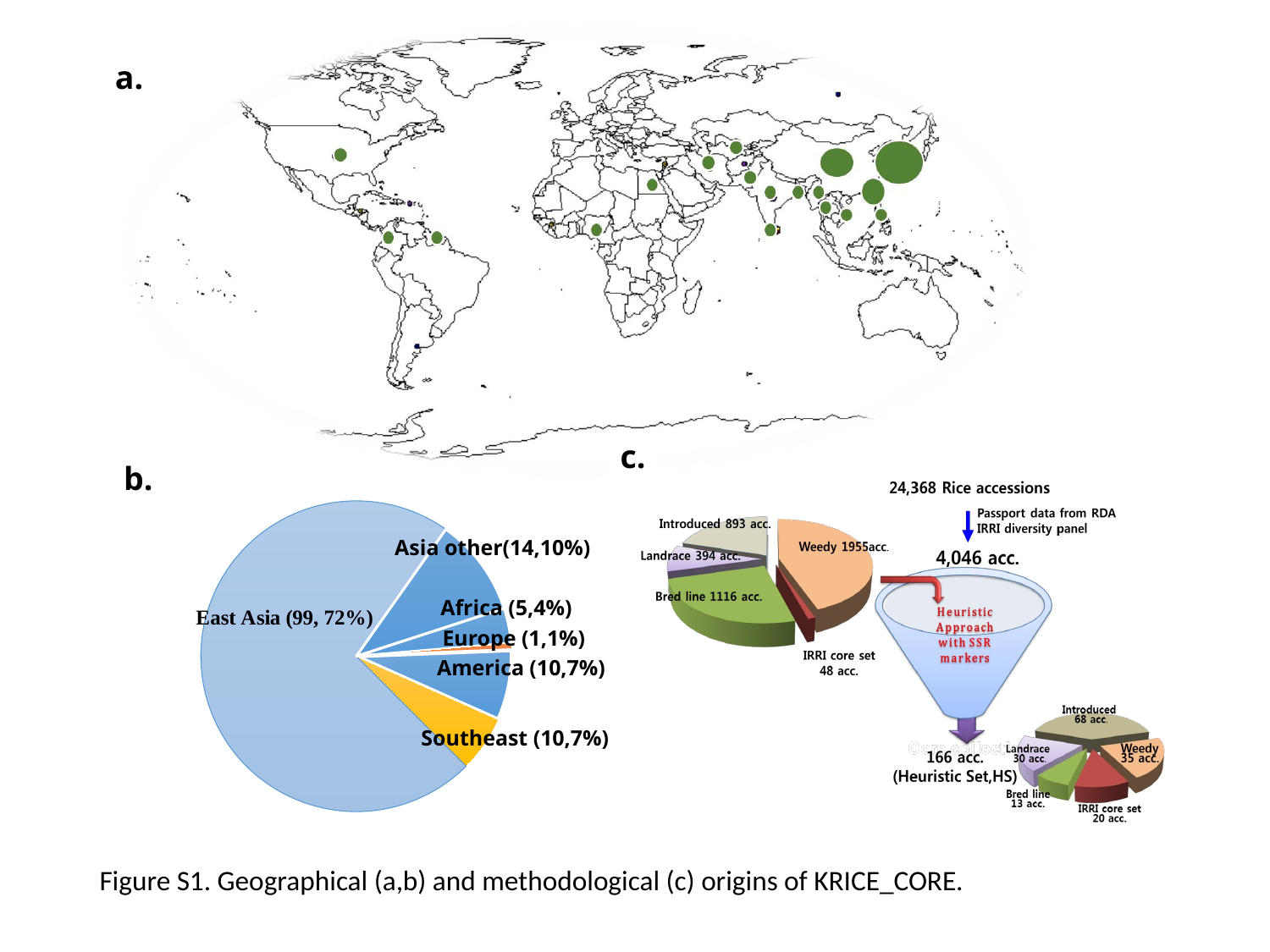

a.
c.
b.
### Chart
| Category | |
|---|---|
| Africa | 5.0 |
| Europe | 1.0 |
| America | 10.0 |
| Southeast Asia | 8.0 |
| east Asia | 99.0 |
| Asia other | 14.0 |Asia other(14,10%)
Africa (5,4%)
Europe (1,1%)
America (10,7%)
Southeast (10,7%)
Figure S1. Geographical (a,b) and methodological (c) origins of KRICE_CORE.

## Slide 2
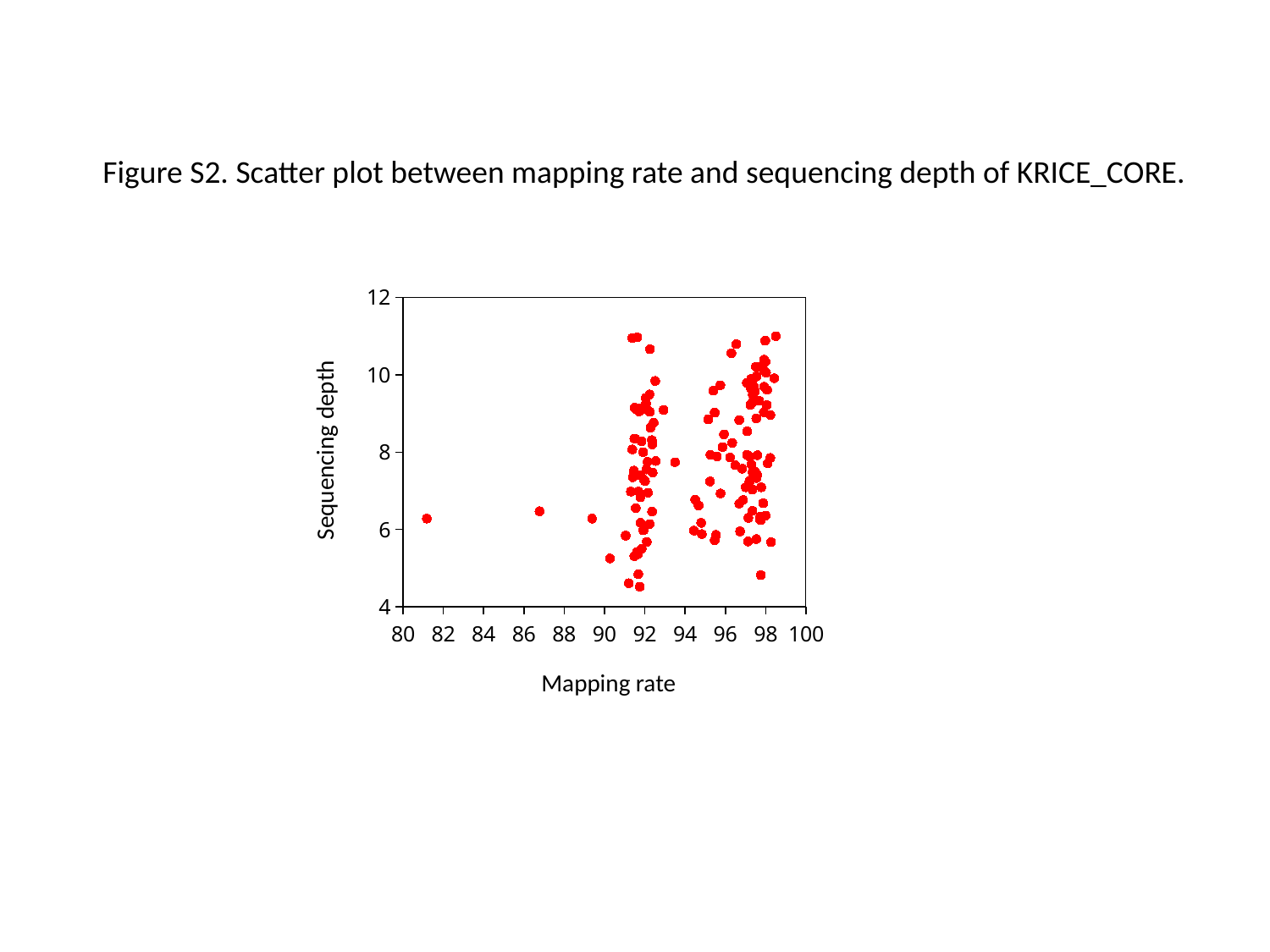

Figure S2. Scatter plot between mapping rate and sequencing depth of KRICE_CORE.
### Chart
| Category | |
|---|---|Sequencing depth
Mapping rate

## Slide 3
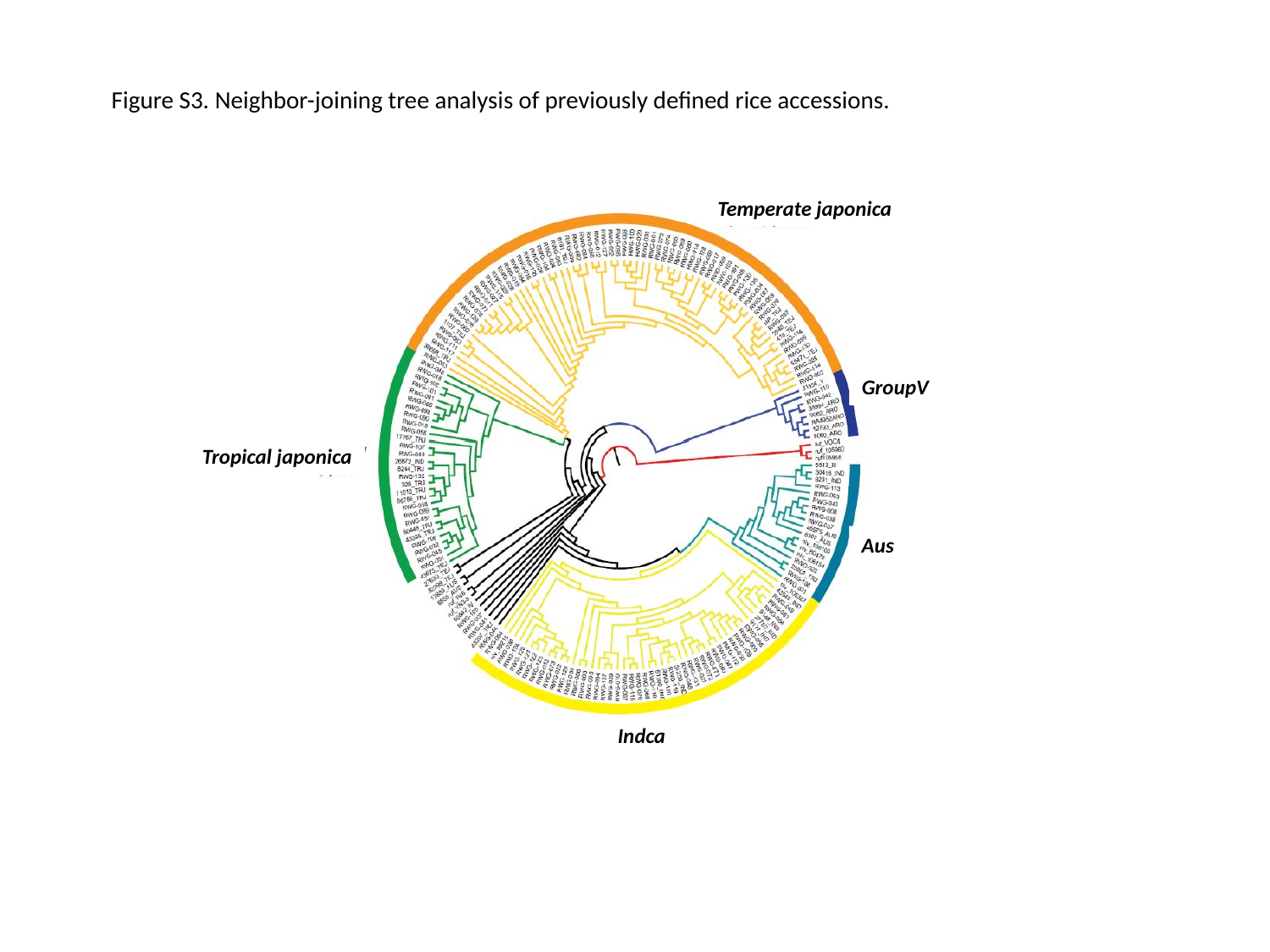

Figure S3. Neighbor-joining tree analysis of previously defined rice accessions.
Temperate japonica
GroupV
Tropical japonica
Aus
Indca

## Slide 4
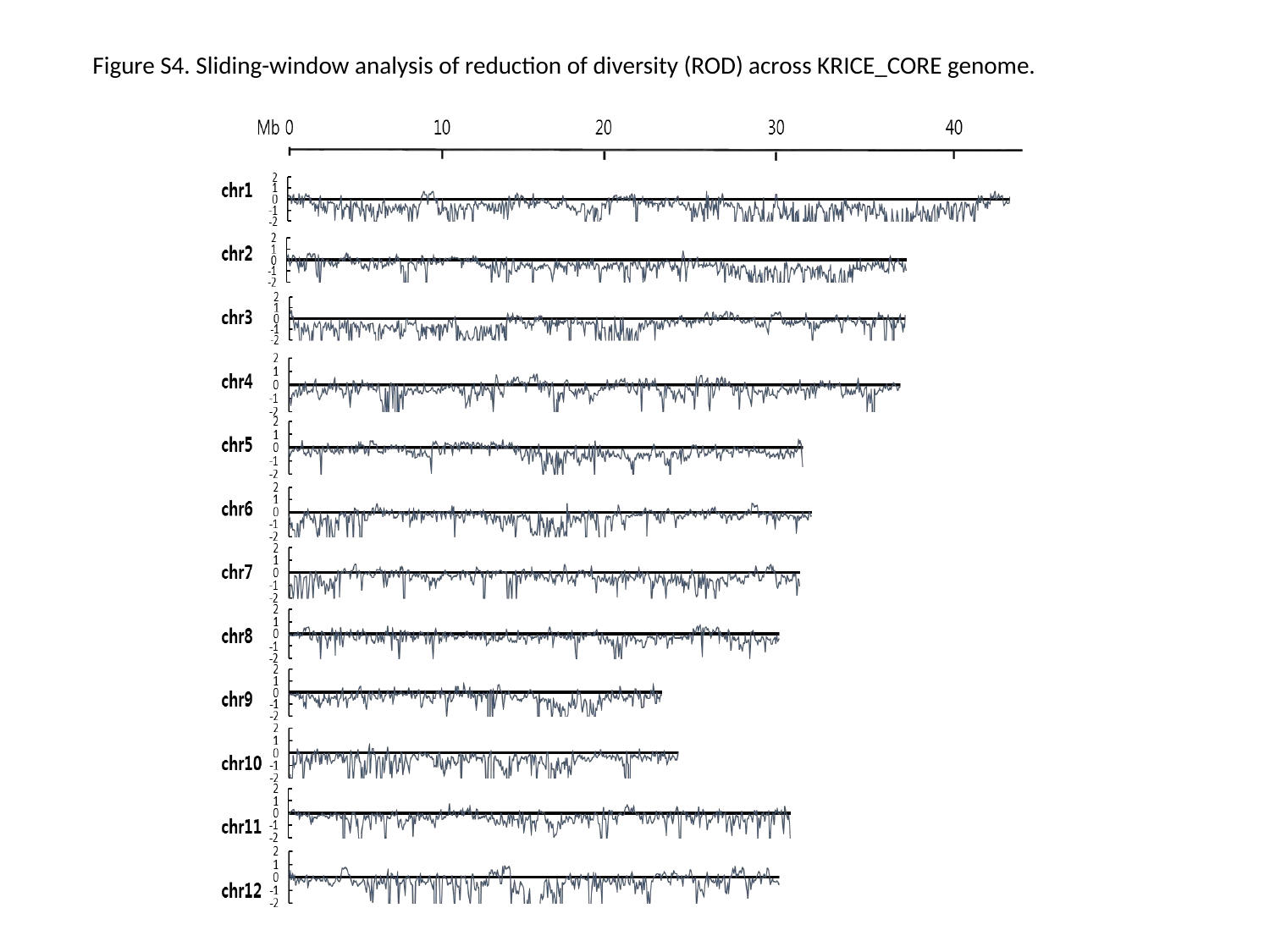

Figure S4. Sliding-window analysis of reduction of diversity (ROD) across KRICE_CORE genome.

## Slide 5
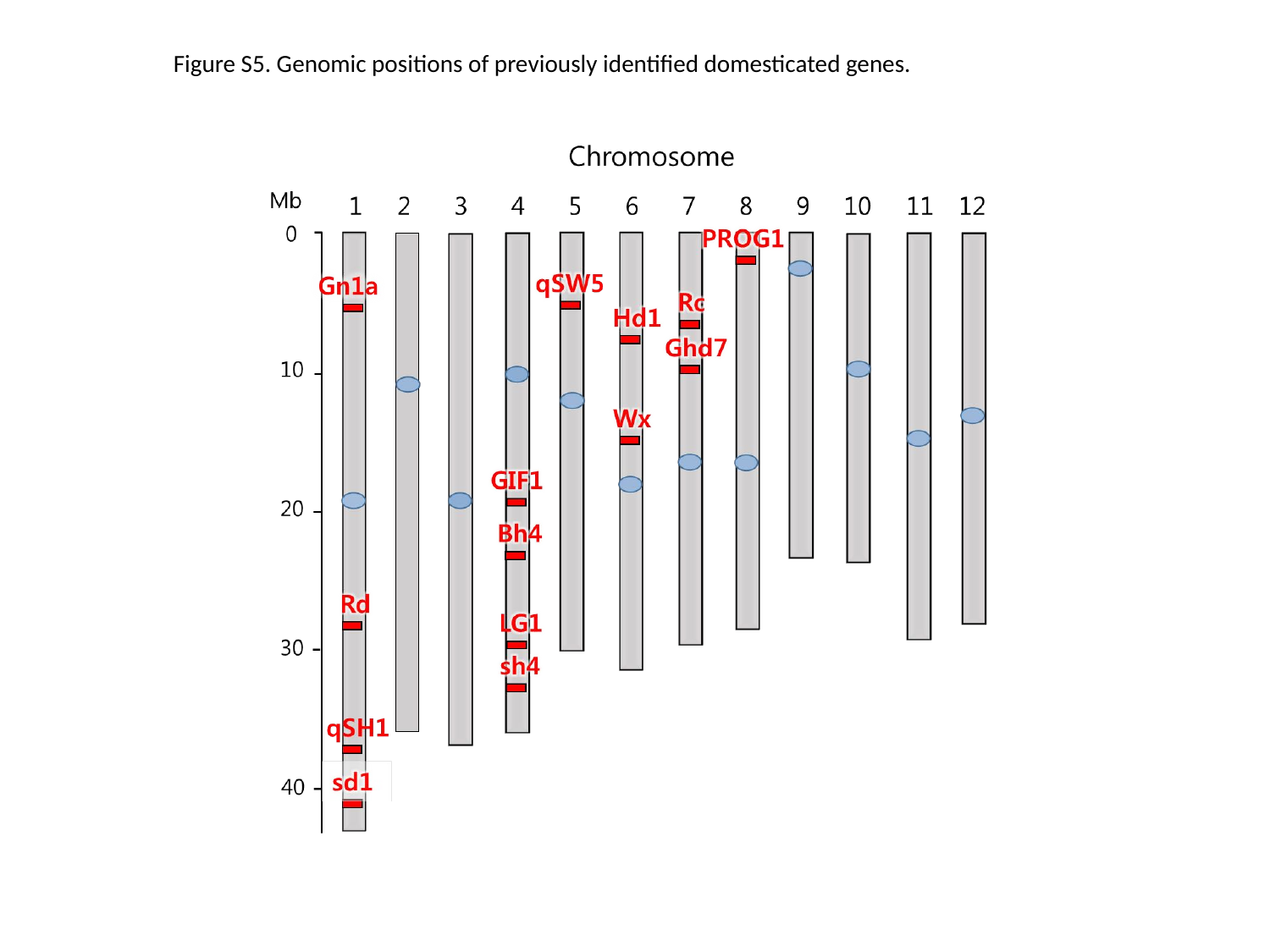

Figure S5. Genomic positions of previously identified domesticated genes.

## Slide 6
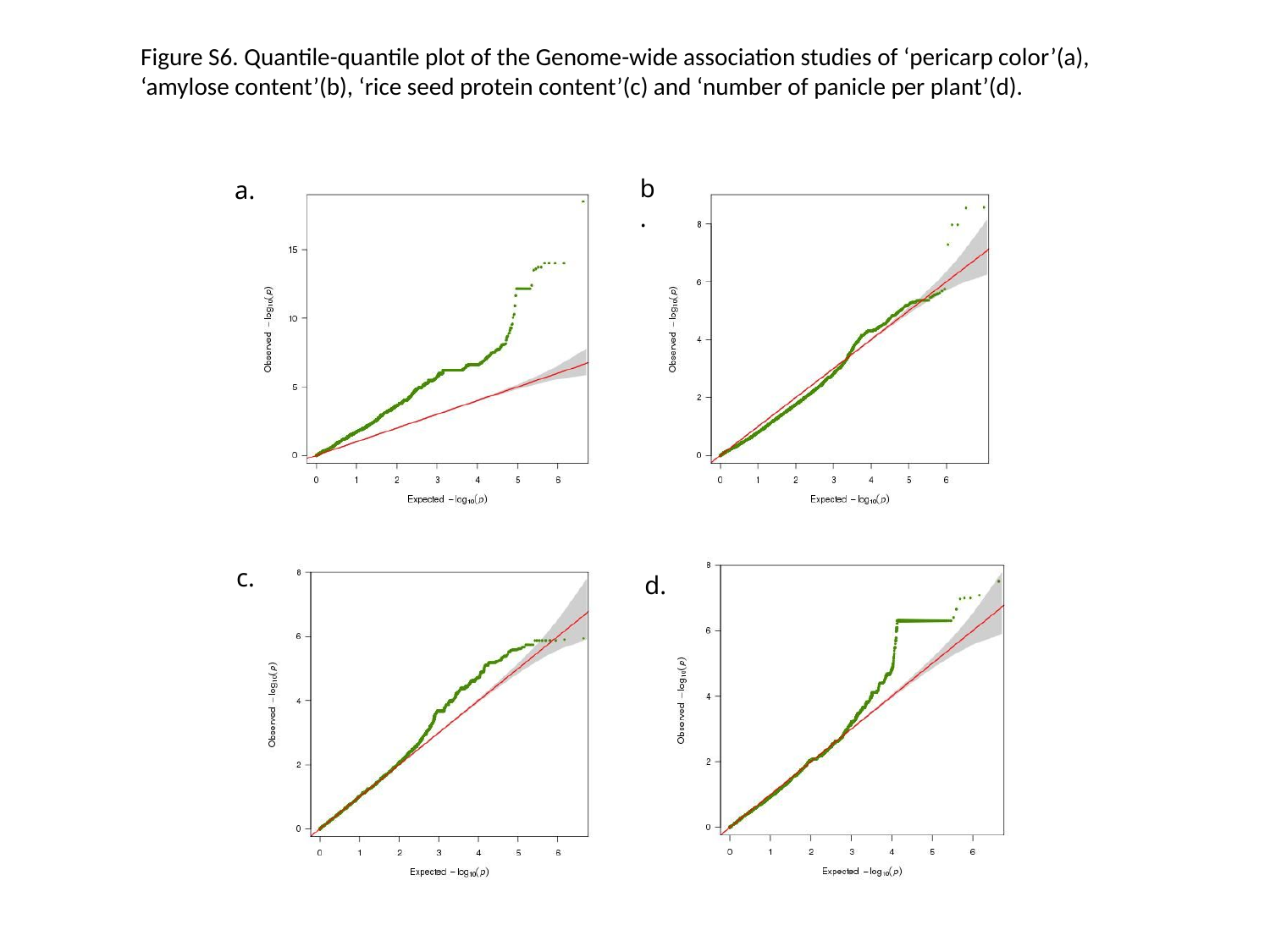

Figure S6. Quantile-quantile plot of the Genome-wide association studies of ‘pericarp color’(a), ‘amylose content’(b), ‘rice seed protein content’(c) and ‘number of panicle per plant’(d).
b.
a.
c.
d.

## Slide 7
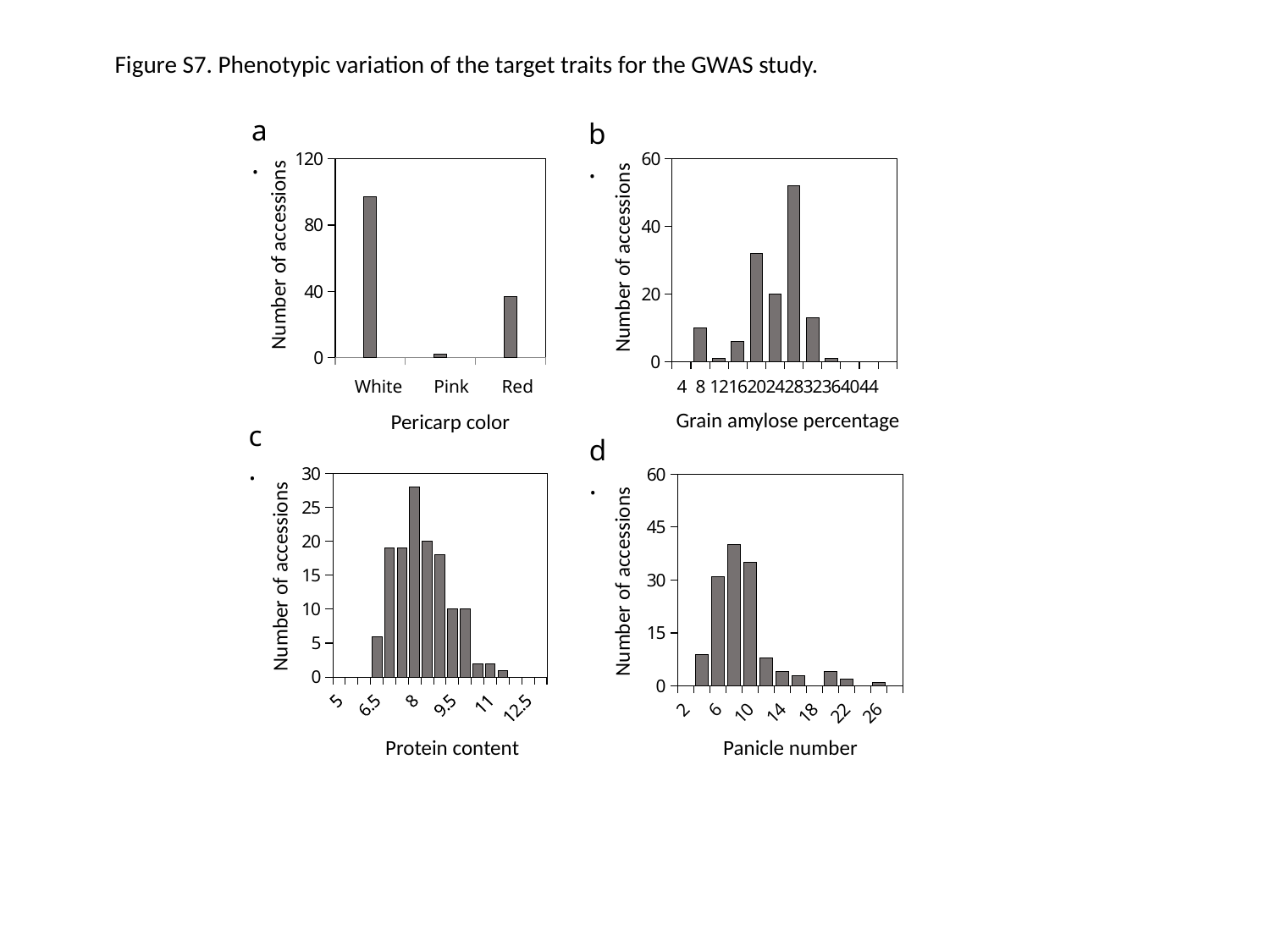

Figure S7. Phenotypic variation of the target traits for the GWAS study.
a.
b.
### Chart
| Category | |
|---|---|
| 1 | 97.0 |
| 1.5 | 2.0 |
| 2 | 37.0 |
### Chart
| Category | |
|---|---|
| 4 | 0.0 |
| 8 | 10.0 |
| 12 | 1.0 |
| 16 | 6.0 |
| 20 | 32.0 |
| 24 | 20.0 |
| 28 | 52.0 |
| 32 | 13.0 |
| 36 | 1.0 |
| 40 | 0.0 |
| 44 | 0.0 |
| | 0.0 |Number of accessions
Number of accessions
Pink
White
Red
Grain amylose percentage
Pericarp color
c.
d.
### Chart
| Category | |
|---|---|
| 2 | 0.0 |
| 4 | 9.0 |
| 6 | 31.0 |
| 8 | 40.0 |
| 10 | 35.0 |
| 12 | 8.0 |
| 14 | 4.0 |
| 16 | 3.0 |
| 18 | 0.0 |
| 20 | 4.0 |
| 22 | 2.0 |
| 24 | 0.0 |
| 26 | 1.0 |
| 28 | 0.0 |
### Chart
| Category | |
|---|---|
| 5 | 0.0 |
| 5.5 | 0.0 |
| 6 | 0.0 |
| 6.5 | 6.0 |
| 7 | 19.0 |
| 7.5 | 19.0 |
| 8 | 28.0 |
| 8.5 | 20.0 |
| 9 | 18.0 |
| 9.5 | 10.0 |
| 10 | 10.0 |
| 10.5 | 2.0 |
| 11 | 2.0 |
| 11.5 | 1.0 |
| 12 | 0.0 |
| 12.5 | 0.0 |
| 13 | 0.0 |Number of accessions
Number of accessions
Panicle number
Protein content
