# Supplementary material for: Genome-wide resequencing of KRICE_CORE reveals their potential for future breeding, as well as functional and evolutionary studies in the post-genomic era
Source: BMC Genomics. 2016 May 26;17:408. doi: 10.1186/s12864-016-2734-y (PMC4882841; doi:10.1186/s12864-016-2734-y)
Supplement: Additional file 5: Table S3. — SNP distributions across various KRICE_CORE genomic regions. Table S4. INDEL distributions across various KRICE_CORE genomic regions. Table S5. Functional categories enriched in the high SNP/INDEL region across the KRICE_CORE genomes. (PPTX 54 kb) [file 12864_2016_2734_MOESM5_ESM.pptx]

## Slide 1
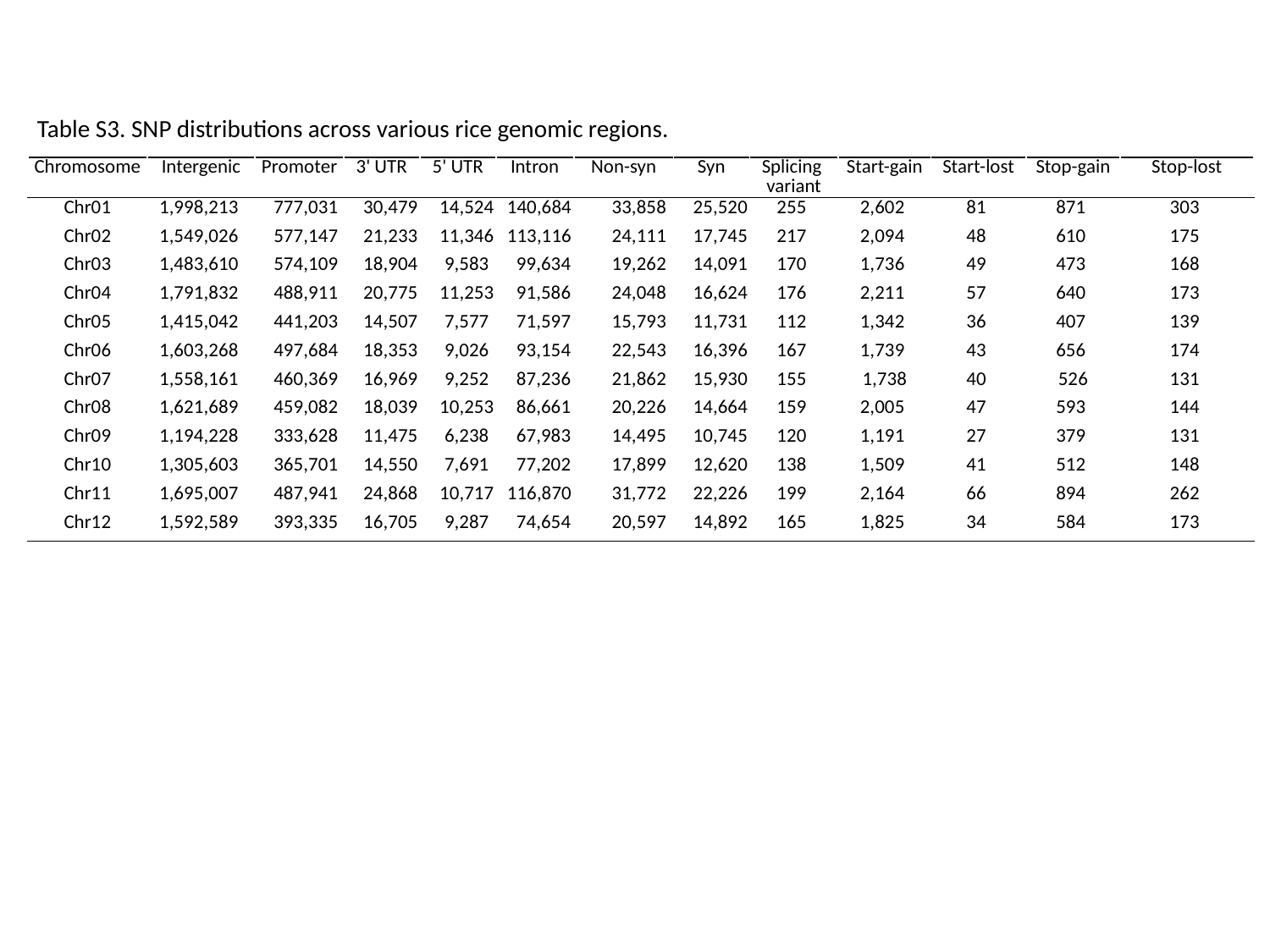

Table S3. SNP distributions across various rice genomic regions.
| Chromosome | Intergenic | Promoter | 3' UTR | 5' UTR | Intron | Non-syn | Syn | Splicing variant | Start-gain | Start-lost | Stop-gain | Stop-lost |
| --- | --- | --- | --- | --- | --- | --- | --- | --- | --- | --- | --- | --- |
| Chr01 | 1,998,213 | 777,031 | 30,479 | 14,524 | 140,684 | 33,858 | 25,520 | 255 | 2,602 | 81 | 871 | 303 |
| Chr02 | 1,549,026 | 577,147 | 21,233 | 11,346 | 113,116 | 24,111 | 17,745 | 217 | 2,094 | 48 | 610 | 175 |
| Chr03 | 1,483,610 | 574,109 | 18,904 | 9,583 | 99,634 | 19,262 | 14,091 | 170 | 1,736 | 49 | 473 | 168 |
| Chr04 | 1,791,832 | 488,911 | 20,775 | 11,253 | 91,586 | 24,048 | 16,624 | 176 | 2,211 | 57 | 640 | 173 |
| Chr05 | 1,415,042 | 441,203 | 14,507 | 7,577 | 71,597 | 15,793 | 11,731 | 112 | 1,342 | 36 | 407 | 139 |
| Chr06 | 1,603,268 | 497,684 | 18,353 | 9,026 | 93,154 | 22,543 | 16,396 | 167 | 1,739 | 43 | 656 | 174 |
| Chr07 | 1,558,161 | 460,369 | 16,969 | 9,252 | 87,236 | 21,862 | 15,930 | 155 | 1,738 | 40 | 526 | 131 |
| Chr08 | 1,621,689 | 459,082 | 18,039 | 10,253 | 86,661 | 20,226 | 14,664 | 159 | 2,005 | 47 | 593 | 144 |
| Chr09 | 1,194,228 | 333,628 | 11,475 | 6,238 | 67,983 | 14,495 | 10,745 | 120 | 1,191 | 27 | 379 | 131 |
| Chr10 | 1,305,603 | 365,701 | 14,550 | 7,691 | 77,202 | 17,899 | 12,620 | 138 | 1,509 | 41 | 512 | 148 |
| Chr11 | 1,695,007 | 487,941 | 24,868 | 10,717 | 116,870 | 31,772 | 22,226 | 199 | 2,164 | 66 | 894 | 262 |
| Chr12 | 1,592,589 | 393,335 | 16,705 | 9,287 | 74,654 | 20,597 | 14,892 | 165 | 1,825 | 34 | 584 | 173 |

## Slide 2
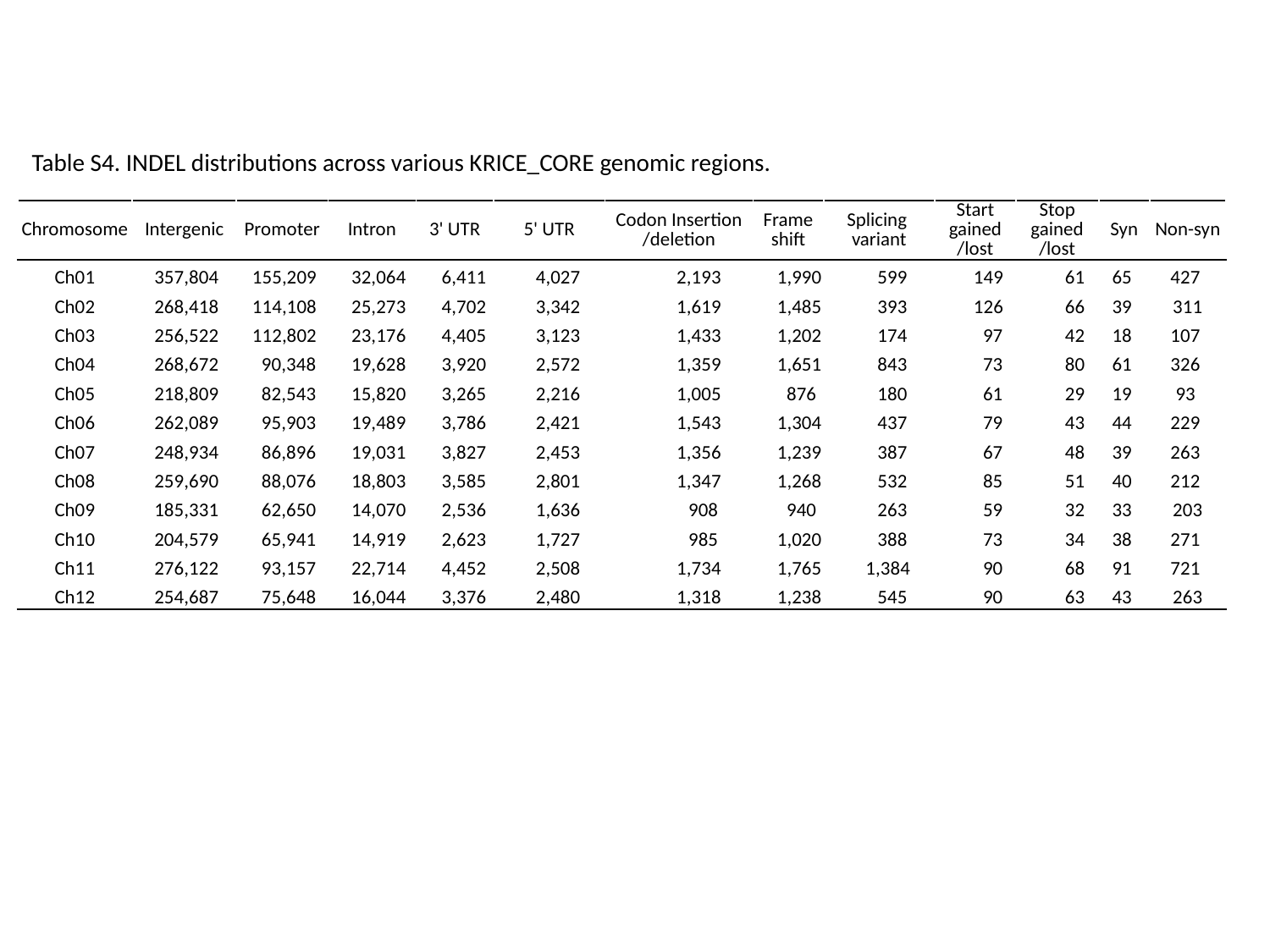

Table S4. INDEL distributions across various KRICE_CORE genomic regions.
| Chromosome | Intergenic | Promoter | Intron | 3' UTR | 5' UTR | Codon Insertion /deletion | Frame shift | Splicing variant | Start gained /lost | Stop gained /lost | Syn | Non-syn |
| --- | --- | --- | --- | --- | --- | --- | --- | --- | --- | --- | --- | --- |
| Ch01 | 357,804 | 155,209 | 32,064 | 6,411 | 4,027 | 2,193 | 1,990 | 599 | 149 | 61 | 65 | 427 |
| Ch02 | 268,418 | 114,108 | 25,273 | 4,702 | 3,342 | 1,619 | 1,485 | 393 | 126 | 66 | 39 | 311 |
| Ch03 | 256,522 | 112,802 | 23,176 | 4,405 | 3,123 | 1,433 | 1,202 | 174 | 97 | 42 | 18 | 107 |
| Ch04 | 268,672 | 90,348 | 19,628 | 3,920 | 2,572 | 1,359 | 1,651 | 843 | 73 | 80 | 61 | 326 |
| Ch05 | 218,809 | 82,543 | 15,820 | 3,265 | 2,216 | 1,005 | 876 | 180 | 61 | 29 | 19 | 93 |
| Ch06 | 262,089 | 95,903 | 19,489 | 3,786 | 2,421 | 1,543 | 1,304 | 437 | 79 | 43 | 44 | 229 |
| Ch07 | 248,934 | 86,896 | 19,031 | 3,827 | 2,453 | 1,356 | 1,239 | 387 | 67 | 48 | 39 | 263 |
| Ch08 | 259,690 | 88,076 | 18,803 | 3,585 | 2,801 | 1,347 | 1,268 | 532 | 85 | 51 | 40 | 212 |
| Ch09 | 185,331 | 62,650 | 14,070 | 2,536 | 1,636 | 908 | 940 | 263 | 59 | 32 | 33 | 203 |
| Ch10 | 204,579 | 65,941 | 14,919 | 2,623 | 1,727 | 985 | 1,020 | 388 | 73 | 34 | 38 | 271 |
| Ch11 | 276,122 | 93,157 | 22,714 | 4,452 | 2,508 | 1,734 | 1,765 | 1,384 | 90 | 68 | 91 | 721 |
| Ch12 | 254,687 | 75,648 | 16,044 | 3,376 | 2,480 | 1,318 | 1,238 | 545 | 90 | 63 | 43 | 263 |

## Slide 3
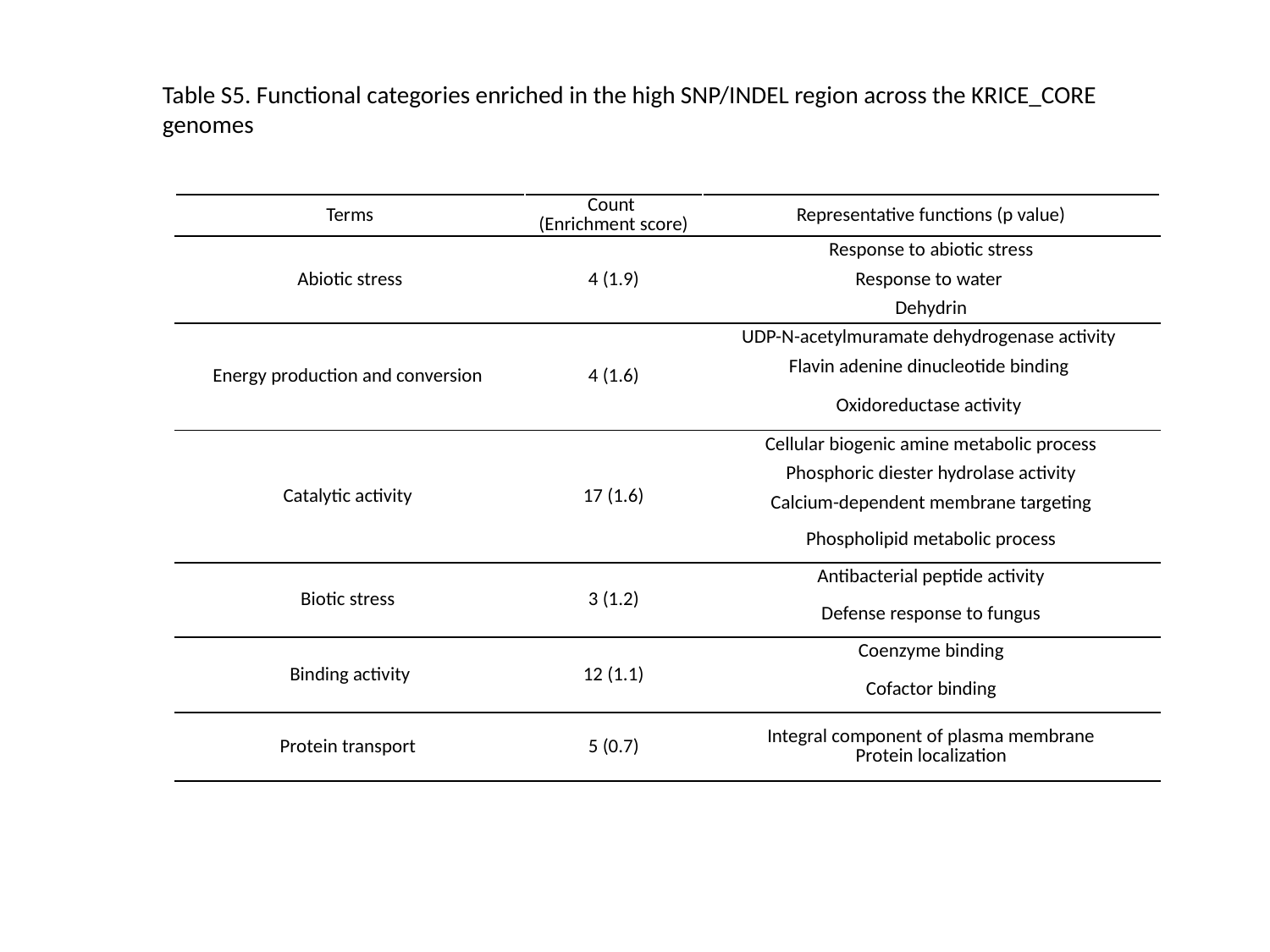

Table S5. Functional categories enriched in the high SNP/INDEL region across the KRICE_CORE genomes
| Terms | Count (Enrichment score) | Representative functions (p value) |
| --- | --- | --- |
| Abiotic stress | 4 (1.9) | Response to abiotic stress |
| | | Response to water |
| | | Dehydrin |
| Energy production and conversion | 4 (1.6) | UDP-N-acetylmuramate dehydrogenase activity |
| | | Flavin adenine dinucleotide binding |
| | | Oxidoreductase activity |
| Catalytic activity | 17 (1.6) | Cellular biogenic amine metabolic process |
| | | Phosphoric diester hydrolase activity |
| | | Calcium-dependent membrane targeting |
| | | Phospholipid metabolic process |
| Biotic stress | 3 (1.2) | Antibacterial peptide activity |
| | | Defense response to fungus |
| Binding activity | 12 (1.1) | Coenzyme binding |
| | | Cofactor binding |
| Protein transport | 5 (0.7) | Integral component of plasma membrane Protein localization |
